# First record of besnoitiosis caused by *Besnoitia bennetti* in donkeys from the UK

**DOI:** 10.1186/s13071-020-04145-8

**Published:** 2020-06-03

**Authors:** Hany M. Elsheikha, Gereon Schares, Georgios Paraschou, Rebekah Sullivan, Richard Fox

**Affiliations:** 1 School of Veterinary Medicine and Science, University of Nottingham, Sutton Bonington, Loughborough, LE12 5RD UK; 2 Friedrich-Loeffler-Institut, Federal Research Institute for Animal Health, Institute of Epidemiology, Greifswald-Insel Riems, Germany; 3 Pathology Department, The Donkey Sanctuary, Brookfield farm, Offwell, Honiton, Devon UK; 4 Veterinary Department, The Donkey Sanctuary, Brookfield farm, Offwell, Honiton, Devon UK; 5 Finn Pathologists, Unit 3c, 3 Mayflower Way, Harleston, Norfolk UK

**Keywords:** Donkey, *Besnoitia bennetti*, Tissue cysts, Histopathology, Specific anti-*Besnoitia* antibodies, Microsatellite typing

## Abstract

**Background:**

The involvement of *Besnoitia bennetti* in skin pathologies was investigated in a series of 20 donkeys from the Donkey Sanctuary in England, in the 2013–2019 period.

**Methods:**

The initial histopathological finding of *Besnoitia* cysts in skin lumps that were presumed to be sarcoids in 2013 triggered our cognisance of this parasite and resulted in identification of a total of 20 cases. Histopathological examination of surgical biopsy samples collected from 8 live donkeys and tissue specimens from 12 deceased donkeys at *post-mortem* examination revealed the presence of *Besnoitia* cysts in all 20 donkeys. The indirect fluorescent antibody test (IFAT) and immunoblotting analysis showed the presence of anti-*Besnoitia* antibodies in archived serum samples from 4 deceased donkeys. Additionally, infection was evidenced in one live donkey based on IFAT and immunoblot analysis of tissue fluid of a dermal mass containing *Besnoitia* cysts, and real-time (RT)-PCR analysis and microsatellite genotyping of DNA isolated from the tissue of the same dermal mass confirmed the infection specifically as *B. bennetti*.

**Results:**

Both serological and microsatellite analyses confirmed the aetiology to be *B. bennetti*. Our findings suggested that in cases of skin masses such as sarcoids, the suspicion of *B. bennetti* infection should be borne in mind even when clinical and histopathology examination results are negative in order to avoid misdiagnosis.

**Conclusions:**

This case series documents, to our knowledge, the first report of *B. bennetti* infection in donkeys in the UK, indicating that donkey besnoitiosis has become noteworthy in the UK. Further investigations of the occurrence, epidemiological characteristics, and clinical manifestations of *B. bennetti* infection in donkeys and other equids are warranted.
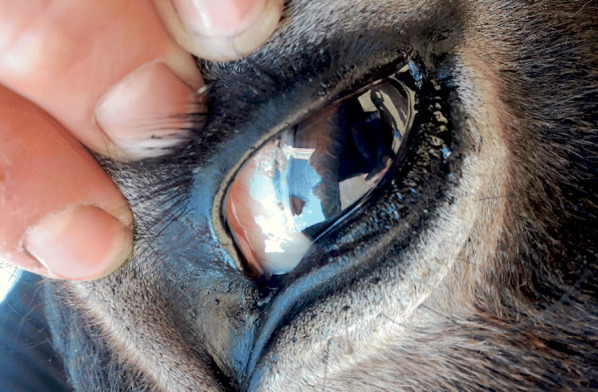

## Background

Besnoitiosis in horses, donkeys, mules and zebras is caused by *Besnoitia bennetti*, which was first described in Sudan in the 1920s. In Europe, this parasite was reported in France in 1922 [1]. *Besnoitia bennetti* infection manifests clinically as multiple, pinpoint, raised, round, yellow to white, up to about 1 mm in diameter cysts in the skin over the head and body, within the nares, on the external and internal pinnae, and on the limbs and perineum. Mucous membranes, such as the ocular limbar sclera (scleral pearls) and the laryngeal mucosa can be also affected [2]. Affected donkeys and horses may be otherwise healthy or present with generalised cachexia and debilitation, similar to that observed in bovine besnoitiosis [3].

The current standard for diagnosing besnoitiosis in donkeys is based on histopathological examination of tissues, which can take several days to produce results and does not allow differentiation between the different *Besnoitia* spp. These limitations have driven the development of more efficient alternative methods to histopathology in order to provide accurate information about the etiological agent. These methods were designed to detect specific *Besnoitia* ribosomal DNA in tissue biopsies using PCR followed by DNA sequencing of the amplicon to identify the species [2, 4, 5] or measure the level of anti-*Besnoitia* spp. specific antibodies in the serum using serological assays such as ELISA, immunofluorescent and immunoblotting analysis [4–9]. Recently, a more specific genotyping method based on microsatellite markers has been developed to distinguish *B. bennetti* from the closely related *B. besnoiti* and *B. tarandi*, which cause besnoitiosis in cattle and reindeer, respectively [10].

Besnoitiosis in donkeys went from a rarely detected parasite to being reported in donkeys in many countries, including the USA [2, 4, 7], Spain, [8], Belgium [5], Italy [9] and Portugal [11]. This increase in the number of reports of *B. bennetti* infection in donkeys and other equids is probably attributed to the increasing awareness of the disease [12]. The emergence of *B. bennetti* in a new geographical region is often associated with a concern in causing invasive infections and the potential of outbreaks in local donkeys [2].

In the present study, we report for the first time in the UK a series of 20 donkeys infected by *B. bennetti*. The initial identification of *Besnoitia* cysts was performed using histopathology and the confirmation of *B. bennetti* was achieved using molecular assays, including microsatellite typing for the first time in Europe. The implications of the research findings are discussed.

## Methods

### Clinical history

The present study involved 20 donkeys infected by *Besnoitia*. The donkeys never lived outside the UK, except for one donkey which originated from Ireland. All donkeys in this study were residents of the UK Donkey Sanctuary, kept on various sites in East Devon and a single location in Dorset. The initial case was found incidentally in 2013, when an adult gelding was presented for surgical excision of a small nodular intradermal mass from the periorbital region. The mass was presumed to be a sarcoid; however, histopathology revealed that the intradermal mass consisted of *Besnoitia* cysts, with no evidence of sarcoid. Subsequent to this case, others in this series presenting with skin masses were also found to have *Besnoitia* cysts. Since then, *Besnoitia* cysts have become a routine differential diagnosis for skin masses by the local veterinary team. A growing awareness of other potential symptoms of the parasite, including scleral and conjunctival lesions, enabled, for example, the case with an ocular presentation to be correctly diagnosed.

Between February and December 2019, eight of the donkeys from the series, which were still alive, were clinically examined, either by the author (RS) or by other veterinary surgeons from the Veterinary Team at The Donkey Sanctuary. This included checking their clinical records, in order to establish any disease since *Besnoitia* cysts were identified. A complete physical examination with particular attention made of the skin, conjunctiva and sclera, was conducted in order to assess the development of any new lesions. A routine oral exam to check the oropharynx and larynx was not carried out; however, all donkeys received routine dental checks every 3–12 months and no lesions were recorded by the attending veterinarian or equine dental technician. Additionally, the lips, rostral tongue and buccal surfaces were visually inspected in July 2019 with respect to cases 2, 4, 5, 6 and 7.

### Histopathological examination

Surgical biopsy specimens of the pinpoint nodules found on the conjunctiva or sclera of the eyes, or of the presumed sarcoid haired skin masses were obtained from 8 live donkeys. Additional samples were obtained from lesions observed in 12 other cases during a routine *post-mortem* examination (PM). Of these 12 donkeys, 8 donkeys were found to have skin masses during PM examination, 2 had presented with laminitis and 2 donkeys had presented with skin masses during *ante-mortem* clinical examination. These latter 2 donkeys were subsequently euthanized due to inoperable or recurrent sarcoids. All collected samples (PM samples and surgical biopsies) were fixed in 10% neutral buffered formalin, processed routinely, embedded in paraffin-wax, sectioned and stained with haematoxylin and eosin (H&E). Mite infestation and other viral, bacterial and fungal agents were ruled out on the basis of clinical signs, history, and the absence of characteristic macroscopic and microscopic lesions for those infectious agents.

### Serological analysis

A total of five samples were subjected to serological analysis using indirect fluorescent antibody test (IFAT) and immunoblot analysis. These included one tissue fluid from a dermal nodular mass obtained from one of the live eight cases (donkey no. 8, Table 1) and four archived serum samples that were collected from terminal blood of four donkeys (donkey nos. 3, 4, 5 and 7, Table 2) and kept frozen at − 60 °C. Unfortunately, due to the retrospective nature of the study, serum samples were only available in this small subset of cases, preventing complete assessment of *Besnoitia* seropositivity in all 20 donkeys. The Bb1Evora03 strain of *B. besnoiti* was maintained in MARC-145 cell culture and extracellular tachyzoites were purified as previously described [10]. Purified tachyzoites were used to prepare IFAT slides or were pelleted by centrifugation at 1300×*g* for 10 min and frozen at − 80 °C until used for immunoblotting. Bradyzoites were purified from lichenified and partially alopecic skin samples collected after slaughtered *B. besnoiti*-infected cattle from Herd-BbGer1, the herd in which besnoitiosis was detected in Germany the first time. These tissues had been frozen at − 20 °C and submitted to the Friedrich-Loeffler-Institut, Germany. Purification was performed as previously described [10]. Eluted parasites were concentrated by centrifugation at 1300×*g* for 10 min, and frozen at − 80 °C until used for immunoblotting.

**Table 1:** Summary of donkeys with besnoitiosis presented clinically as skin or ocular masses

ID	Age and sex	Year	Clinical history	Lesions^d^
1^c^	17-year-old gelding	2013	Dermatitis and skin masses in various sites of body	Small nodules on skin of left eye periorbital area
2^c^	5-year-old gelding	2016	Lumps on the lip	Nodular mass on the penile sheath skin
3^c^	5-year-old gelding	2018	Nodular masses in right hand side of abdomen and sheath skin	Nodular mass on the penile sheath skin
4^c^	7-year-old gelding	2018	Recurrent sarcoids	Nodular masses in skin overlying left mandible
5^c^	11-year-old gelding	2018	Previously removed mass in skin overlying the left mandible. Recurrent sarcoids in various sites treated with chemotherapy	Mass on the lip
6^b^	5-year-old female	2018	Corneal ulcer in the eye	Nodules noticed on sclera (Fig. 1). *Besnoitia* cysts in conjunctiva
7^c^	7-year-old gelding	2019	Previously removed mass (surgical excision with cutting laser 2016) in skin overlying left mandible	Nodular sarcoid mass in skin overlying left mandible
8^a,c^	20-year-old gelding	2019	Episodes of conjunctivitis and weight loss	Small mass at the base of right teat and lumps on sheath skin

^a^*Besnoitia bennetti* infection was confirmed in a tissue sample by PCR and the tissue fluid of the same sample was reactive to *B. besnoiti* bradyzoites based on immunoblot results

^b^*Besnoitia* cysts were associated with ocular lesions

^c^Nodular masses were removed by surgical excision with a cutting laser (except for donkey no. 1 where surgery was performed using scalpel excision as the hospital did not have access to a surgical laser at this time). Histopathology of the excised mass revealed *Besnoitia* cysts adjacent to the sarcoid tissue in all donkeys, except for donkey no. 1 where only *Besnoitia* cysts were detected, without any sarcoid neoplasm as was initially suspected and donkey no. 3 where the histopathology was suggestive, but not definitive of sarcoid for the flank mass and no sarcoid tissue was identified in the penile sheath mass

^d^Histological examination of surgical biopsies from the 8 donkeys identified 1 to 3 *Besnoitia* cysts per biopsy surrounded by mild to moderate number of lymphocytes and/or eosinophils

**Table 2:** Summary of findings in 12 deceased donkeys with besnoitiosis identified over a period of 3 years (from 2016 to 2019)

ID	Age and sex	Year	Clinical history	Pathological findings
1^a^	29-year-old female	2016	Chronic stiffness, osteoarthritis and alopecia	Hyperkeratosis, ulceration and lymphoplasmacytic dermatitis, with one *Besnoitia* cyst in the dermis
2^a^	28-year-old gelding	2016	Chronic and progressive alopecia, crusts and ulcers	Multiple eosinophilic granulomas and *Besnoitia* cysts in the dermis with no associated inflammation
3^a,c^	6-year-old gelding	2016	Dental disease, penile sheath phimosis and skin issues	*Besnoitia* cysts were found within the dermis with no surrounding inflammation
4^a,c^	5-year-old gelding	2017	Repeated sarcoids plus an inoperable sarcoid on penile sheath	Moderate enlargement of inguinal lymph nodes with one *Besnoitia* cyst in the subcapsular space of the inguinal lymph node
5^c^	28-year-old gelding	2017	Intractable pain, corneal ulceration, keratitis and conjunctivitis	Chronic, mild to moderate, conjunctivitis with intralesional *Besnoitia* cysts in both conjunctivae
6	23-year-old gelding	2017	Severe dental disease and chronic osteoarthritis	Chronic, moderate, diffuse lymphoplasmacytic conjunctivitis with one *Besnoitia* cyst in the conjunctiva and eyelid
7^b,c^	8-year-old gelding	2018	Inoperable sarcoid mass in the left-hand side of the upper lip	*Besnoitia* cysts were identified adjacent to sarcoid without associated inflammation
8	32-year-old gelding	2018	Lameness due to hoof abscess, with stiffness and arthritis	*Besnoitia* cysts in iris, extraocular muscles, conjunctiva, sclera, third eyelid and larynx
9^b^	13-year-old gelding	2019	Recurrent sarcoids and neurological signs	*Besnoitia* cysts were identified adjacent to a sarcoid without associated inflammation
10^a^	21-year-old female	2019	Chronic and progressive lameness and osteitis	*Besnoitia* cysts surrounded by lymphoplasmacytic inflammation in laminar corium of the left fore limb
11^a^	24-year-old female	2019	Chronic laminitis, hoof abscess and third phalanx osteitis	*Besnoitia* cyst surrounded by inflammation in the third phalanx and stratum lamellatum of the right fore limb
12	28-year-old gelding	2019	Recurrent, persistent ulcerative dermatitis	Chronic, multifocal, lymphoplasmacytic inflammation with intralesional *Besnoitia* cysts in the larynx, muzzle, sclera of both eyes and conjunctiva

^a^Cases with dermatological involvement (i.e. without grossly detectable eye or upper respiratory lesions) were diagnosed by histopathology

^b^Donkeys were diagnosed with sarcoid *ante-mortem*, but later on died due to non-parasitic reasons and *Besnoitia* cysts were detected during histopathology examination

^c^Diagnosis was confirmed in 4 archived serum samples based on a positive IFAT cut-off titre of 1:400 and recognition of > 3 of 10 bands in the immunoblots

#### Indirect fluorescent antibody test (IFAT)

Serial dilutions starting at 1:50 of donkey sera were analyzed for *Besnoitia* antibodies by IFAT using fluorescein (FITC)-conjugate solution [affinity purified goat anti-horse IgG (H + L) (Jackson ImmunoResearch Laboratories, West Grove, USA; diluted 1:50 in PBS)]. The procedure was performed as previously described [10]. Sera from infected or non-infected donkeys were used as positive and negative controls, respectively, sampled during a besnoitiosis outbreak in north-eastern Pennsylvania [7].

#### Immunoblot analysis

Immunoblots were performed as described previously [10]. Samples containing 2 × 10^6^*Besnoitia* tachyzoites/lane were treated for 10 min at 94 °C with non-reducing sample buffer (2% (w/v) SDS, 10% (v/v) glycerol, 62 mM Tris HCl, pH 6.8). The parasite samples were electrophoresed in a 12.5% (w/v) SDS-polyacrylamide minigel together with marker proteins (LMW-SDS Marker Kit, GE Healthcare, Germany). Separated parasite antigens and marker proteins were electrophoretically transferred to polyvinylidene fluoride (PVDF) membranes (Immobilon-P, Millipore, Darmstadt, Germany) in a semidry transfer system, using a current of 1.5 mA/cm^2^ gel for 90 min. The part of the membrane coated with the marker proteins and a 0.5 mm wide strip of the antigen-coated part was cut-off and the transferred proteins were visualized using an India ink stain. The remaining antigen-coated membrane was blocked for 30 min at ambient temperature with PBS-TG (PBS, 0.05% (v/v) Tween 20, 2% (v/v) fish gelatine liquid) (Serva, Heidelberg, Germany), air-dried overnight, cut into up to 60 strips, which were stored frozen at − 20 °C. Prior to incubation with diluted serum samples, the strips were blocked again with PBS-TG for 30 min at ambient temperature.

To detect antibodies against parasite antigens, the strips were incubated with serum as previously described [13] with few modifications. Serum samples diluted in PBS-TG were incubated with the strips for 60 min at ambient temperature. Sera were diluted 1:100 with PBS-TG and incubated for 60 min (room temperature). For donkey positive and negative control sera, sera were applied from infected or non-infected donkeys, respectively, sampled during a besnoitiosis outbreak in north-eastern Pennsylvania [7]. After washing in PBS-T (PBS, 0.05% (v/v) Tween 20), the strips were incubated with peroxidase conjugate solution (affinity purified goat anti-horse IgG (H + L) (Jackson ImmunoResearch Laboratories, West Grove, USA) diluted 1:500 in PBS-TG) for 60 min at ambient temperature. After washing in PBS-T and distilled water, antibody reactions were detected by adding substrate solution (40 μl H_2_O_2_ 30% (v/v) and 30 mg 4-chloro-1-naphthol (Sigma-Aldrich, Taufkirchen, Germany) in 40 ml TBS, 20% (v/v) methanol). Relative molecular masses were determined by comparison with the LMW-SDS Marker standard. Reactions to 10 antigen bands were recorded, based on Schares et al. [10]. A positive reaction with at least 4 of the 10 previously selected and described antigenic bands was regarded as a positive immunoblot reaction.

### Molecular characterization

#### DNA isolation and real-time (RT)-PCR

A single skin biopsy, containing tissue cysts, from one donkey (donkey no. 8, Table 1) was dissected into 20 portions of 25 mg each and DNA was extracted using a commercial kit (NucleoSpin® Tissue, Macherey-Nagel, Düren, Germany) according to the manufacturers’ instructions. The RT-PCR, which covers all *Besnoitia* species of ungulates, was performed using the BbRT2 protocol with the primers Bb3 and Bb6 and the probe Bb3-6 (5′-FAM, 3′-BHQ1) as described previously [14].

#### Microsatellite typing

Microsatellite DNA was amplified as previously described [10]. In details, microsatellite DNA sequences (Markers Bt-5, -6, -7, -9, -21) were amplified by PCR using the primer pairs published previously [15]. In case of Bt-20, we employed newly designed primers Bt-20-GSF (5′-GAG ACA CTT GAT GGA GCA ACG-3′) and Bt-20-GSR (5′-TGT GGA TAC CGT GAA CAG CTG-3′). PCR primers were used at a final concentration of 0.5 μM and dNTPs at 250 μM each (Amersham Biosciences, Piscataway, USA). InviTaq DNA polymerase (Bt-5, -6, -7, -9, -21; STRATEC Molecular, Berlin, Germany) or DyNAzyme II DNA polymerase (Bt-20; Finnzymes, Espoo, Finland) was added at 5 or 2 U/25 μl, respectively, with the provided buffer. The reaction mix was supplemented with bovine serum albumin at a concentration of 20 μg/ml. Water PCR Reagent (Sigma-Aldrich, Taufkirchen, Germany) served as a negative control and DNA from cell culture-derived *B. besnoiti* (Bb1Evora03) tachyzoites was used as a positive control, at a concentration of 10 ng/μl.

The reactions were performed in a thermal cycler (Eppendorf Mastercycler, Personal Thermal Cycler, Hannover, Germany) with an initial denaturation step of 95 °C for 5 min, followed by 10 cycles of denaturation (1 min at 95 °C), annealing (1 min at 52 °C with a decrement of 0.5 °C per cycle), 40 cycles of denaturation (1 min at 95 °C), annealing (1 min at 47 °C) and extension (1 min at 72 °C), and a final extension step at 72 °C for 10 min. In case of Bt-20, the initial denaturation step of 95 °C for 5 min was followed by 10 cycles of denaturation (1 min at 95 °C), annealing (1 min at 57 °C with a decrement of 0.5 °C per cycle), 40 cycles of denaturation (1 min at 95 °C), annealing (1 min at 52 °C) and extension (1 min at 72 °C), and a final extension step at 72 °C for 10 min. The amplification products were visualized in 1.5% agarose gels stained with ethidium bromide. A 100 bp DNA ladder (Invitrogen GmbH, Karlsruhe, Germany) was used as a size standard.

Amplification products were cloned into a commercially available vector (pGEM®-T Easy Vector System I, Promega, Mannheim, Germany) and used to transform chemically competent *Escherichia coli* (OneShot TOP10, Thermo Fisher Scientific, Langenselbold, Germany). The transformed *E. coli* were cultivated, the plasmid-DNA collected and sequenced using a BigDye Terminator v1.1 Cycle Seq. Kit and an ABI 3130 capillary sequencer (both, Thermo Fisher Scientific, Langenselbold, Germany).

## Results

### Clinical characteristics

This case series consists of 20 donkeys. Details of the age, sex, characteristic lesion and clinical history of the 8 live cases and the 12 deceased donkeys are summarized in Tables 1 and 2, respectively. Within this case series, visible *Besnoitia* cysts were observed in sclera, conjunctiva or laryngeal mucosa of 4 donkeys during PM examination, whilst 6 donkeys were diagnosed with skin masses during PM examination and *Besnoitia* cysts were identified at histopathology (Table 2). Two other cases (donkey nos. 7 and 9, Table 2) were diagnosed with sarcoids whilst alive, but later on were euthanized due to inoperable or recurrent sarcoids and histopathology examination of biopsy of the sarcoid tissue confirmed the presence of *Besnoitia* cysts adjacent to sarcoids. Additionally, *Besnoitia* cysts were detected in surgical biopsies in 8 donkeys that are still alive.

**Fig. 1:**
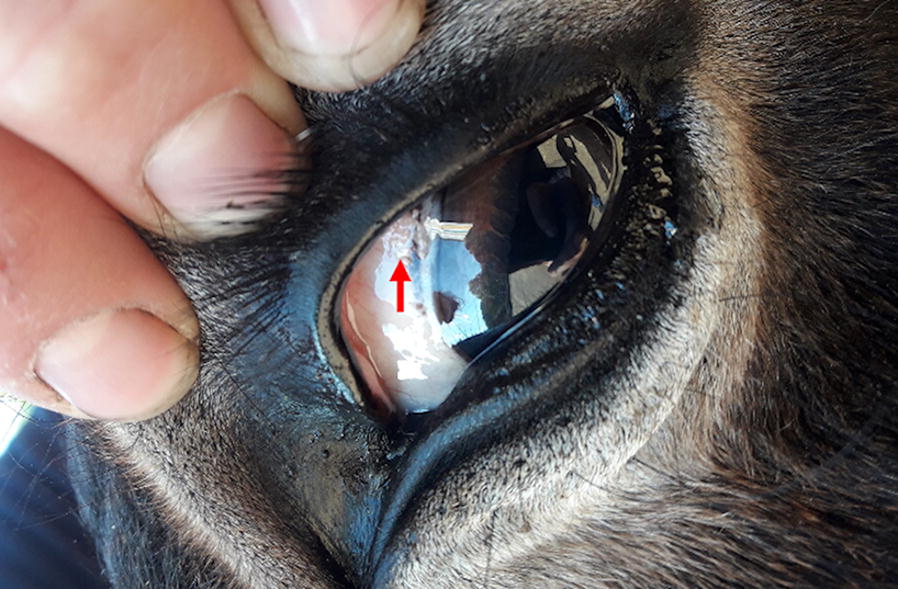
*Besnoitia bennetti* cysts in the sclera of the eye of a 5-year-old donkey (case no. 6, Table 1). The cysts appear as multiple, up to 1 mm in diameter, round, white and firm nodules (arrow)

Those cases presented clinically as skin masses were managed by surgical excision and *Besnoitia* cysts were excised with lesion-free margins. To date, there have been no recurrence of these lesions and surgical excision appears to be locally curative. However, it is worthy to mention that the authors cannot definitively prove or disprove whether *Besnoitia* cysts were present in other locations of the body in these cases. There was a single case with an ocular presentation (donkey no. 6, Table 1), which was presented with ulcerative keratitis, conjunctivitis, epiphora, blepharospasm and chemosis. The affected eye was treated with systemic phenylbutazone 2.2 mg/kg body weight p/o BID for analgesic and anti-inflammatory purposes, in addition to topical application of chloramphenicol and 0.4% hyasent-S (Remend dry eye lubricant drops, Bayer, Leverkusen, Germany). The attending veterinarian noted an unusual pattern of corneal ulceration, which healed rapidly but conjunctival symptoms persisted. At subsequent examination, small nodules were identified as depicted in the photograph presented as Fig. 1. Due to the suspicion that these nodules may represent an eosinophilic keratoconjunctivitis or infection with *Besnoitia*, a biopsy of the lesion was performed. The conjunctival biopsy demonstrated the presence of *Besnoitia* cysts, but the presence of further cysts elsewhere in the conjunctiva has to date, not been excluded. However, there has been no recurrence of clinical signs of keratitis or conjunctivitis.

Clinical checks of the 8 donkeys were performed in early 2019 and 2 donkeys (donkey nos. 3 and 8, Table 1) have had recurrence of their sarcoids but there was no evidence of *Besnoitia* cysts. Other cases have had recurrence of masses in the same location or elsewhere on the body but have not had surgical removal or histopathological analysis. Donkey case 8 was also presented for weight loss investigation in late 2019, alongside a history of corneal ulceration and multiple episodes of white line disease and abscessation causing lameness. The attending veterinarians considered the weight loss to be due to an episode of colitis. This donkey had a nodular lump surgically excised from the right flank in October 2019, histopathology of this mass did not show the presence of any *Besnoitia* cysts and the mass was diagnosed as sarcoid.

### Histological lesions

**Fig. 2:**
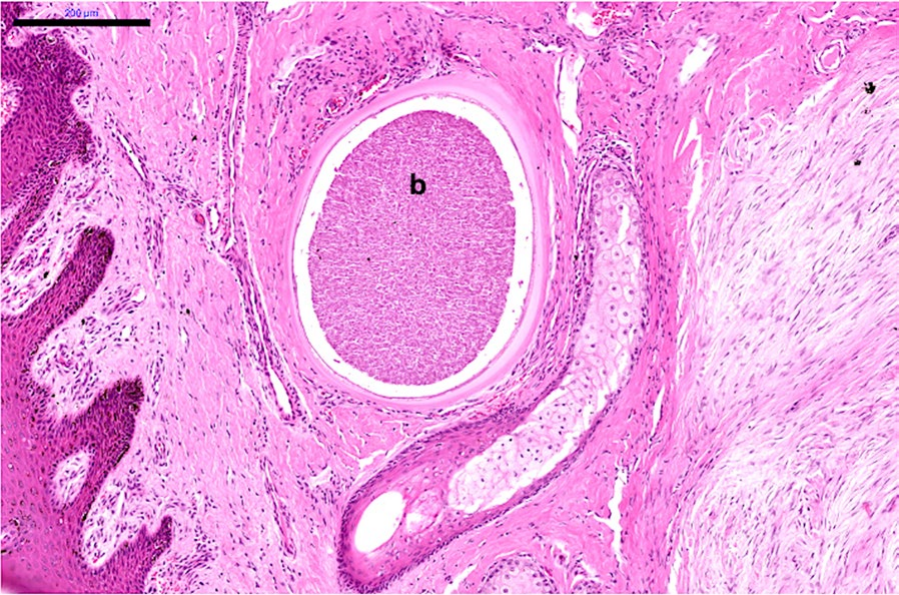
Histopathology of the dermis of a donkey showing the presence of a thick-walled *Besnoitia bennetti* cyst, which is fully packed with bradyzoites (b) and surrounded by mononuclear inflammatory cells, mainly lymphocytes and plasma cells. H&E staining. *Scale-bar*: 200 μm

Examination of the sampled tissues by light microscopy revealed a low to moderate number of spherical to ovoid protozoal cysts that measured 80–450 µm. These cysts were present multifocally within the superficial and/or deep dermis, the submucosa of the larynx, the iris, the submucosa of the conjunctiva, sclera, upper, lower and third eyelid. Mature cysts consisted of an outer, thick, hyalinised, eosinophilic layer, a layer consisting of the cytoplasm of host cells and one or more host cell nuclei and an inner layer that formed the parasitophorous vacuole, which was filled with myriads of tightly packed bradyzoites (Fig. 2).

Some of the cysts were surrounded by low to moderate number of lymphocytes, plasma cells, eosinophils and histiocytes, whereas others were not associated with any inflammation. In seven cases, one or two parasitic cysts were incidentally identified neighbouring tumours that had histopathological features of sarcoids. In seven other cases, *Besnoitia* spp. cysts were identified incidentally in donkeys from samples taken for other reasons. In one case diagnosed with a sarcoid, a single *Besnoitia* cyst and moderate numbers of eosinophils were identified in the subcapsular space and the medullary sinuses of the inguinal lymph node, respectively (presumed drainage from the inflamed *Besnoitia* cysts). In two cases, *Besnoitia* spp. cysts were identified in sections of the ocular sclera, conjunctiva, iris, larynx and muzzle. In one donkey, *Besnoitia* spp. cysts were associated with conjunctivitis. In two donkeys, diagnosed with chronic laminitis, a single *Besnoitia* cyst, surrounded by moderate lymphoplasmacytic inflammation, was detected within the laminar corium and the stratum lamellatum, respectively. Other histopathologic findings of the haired skin that were not associated with the presence of cysts included mild diffuse orthokeratotic hyperkeratosis and mild lymphoplasmacytic and eosinophilic dermatitis.

### Serological findings

**Fig. 3:**
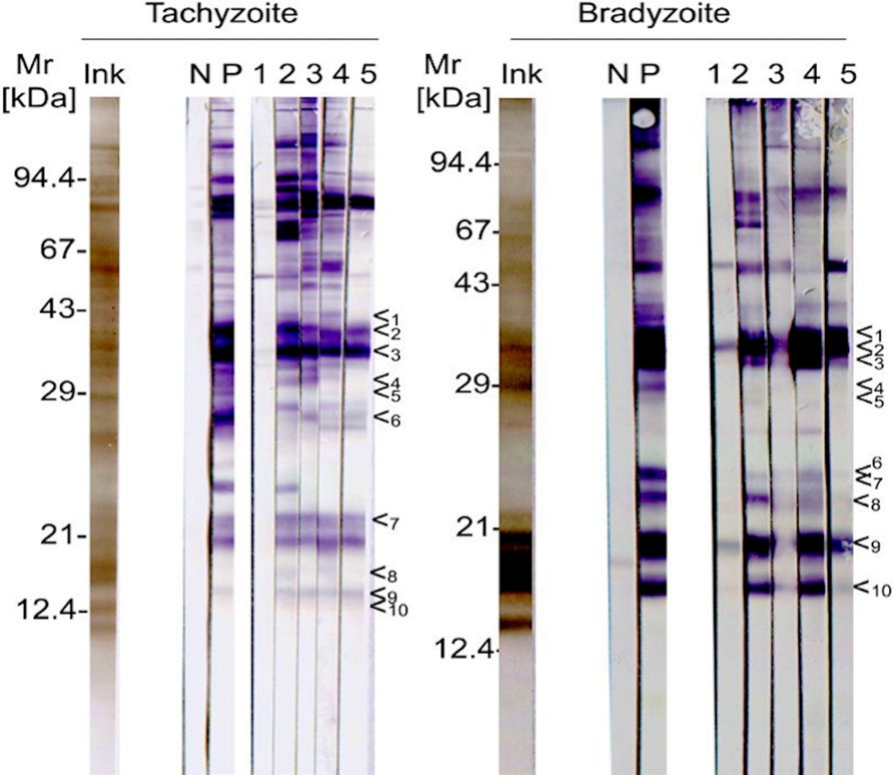
Immunoblot reactions against non-reduced *Besnoitia besnoiti* tachyzoite or bradyzoite antigens of donkeys from UK suspected of being infected with *Besnoitia bennetti*. Antigen bands selected for scoring the immunoblot reactions are marked (<). Ten tachyzoite and 10 bradyzoite antigen bands were recorded due to the absence of cross-reactivity to *Sarcocystis* spp., *N. caninum* or *T. gondii* [10]. Apart from one sample (tissue fluid, donkey no. 8, tachyzoite antigen), all tested samples showed positive reactions with more than 3 of the selected 10 antigen bands (see text for details on the number of recorded bands per sample). Lane 1: tissue fluid from donkey no. 8 (Table 1); Lanes 2–5: sera from donkey nos. 3, 4, 5 and 7 (Table 2). *Abbreviations*: Mr, relative molecular weight; N, negative; P, positive control donkey sera obtained from a previous study in the USA [7]

Archived serum samples from four donkeys (donkey nos. 3, 4, 5 and 7, Table 2), in addition to a tissue fluid sample obtained from one of the live eight clinical cases (donkey no. 8, Table 1), were examined by *B. besnoiti* IFAT assay. All four archived serum samples were positive at 1:400 titre; however, the result of the tissue fluid sample was negative. The same five samples were also examined by *B. besnoiti* immunoblots (using 10 tachyzoite and 10 bradyzoite antigen bands). The sera revealed clear positive results for both tachyzoite and bradyzoite antigens [donkey 3 (T:8; B:7); donkey 4 (T:7; B:5); donkey 5 (T:8; B:8); donkey 7 (T:6; B:8)], where T and B denote the number of reactive tachyzoite and bradyzoite antigen bands, respectively. The tissue fluid sample, however, demonstrated positivity (more than 3 specific bands recognized) only in the bradyzoite immunoblot (Fig. 3).

### Molecular findings

#### Real-time (RT)-PCR results

Individual DNAs of several skin portions obtained from one donkey (donkey no. 8, Table 1) were examined by RT-PCR (BbRT2) assay. All samples tested positive, but to a variable extent. Quantification cycle (Cq) values ranged from 22.7 to 42.9 (median 31.4).

#### Microsatellite typing

DNA with lowest Cq value was used to amplify microsatellite marker regions. This analysis revealed microsatellite patterns characteristic for *B. bennetti* with one exception. In case of Bt-7, *B. bennetti* DNA from donkey in the present study revealed nine repeats, whereas for North American *B. bennetti* eight repeats had been previously reported (Table 3).

**Table 3:** Microsatellite typing of *Besnoitia bennetti* obtained from skin lesion of donkeys according to the number of repeat motifs in six microsatellite loci

*Besnoitia* species	Country/Region	Microsatellite locus						Reference
		Bt-5	Bt-6	Bt-7	Bt-9	Bt-20	Bt-21	
*B. besnoiti*	Germany, Bavaria	10	12	8	10	8	13	[10]
*B. tarandi*	Canada, Quebec	11	12	9	11	11	23	[10]
*B. bennetti*	USA, Texas	12	13	8	8	8	6	[7]
	USA, Michigan	12	13	8	8	8	6	[2, 4]
	UK, England	12	13	9	8	8	6	Present study

*Note*: The sample analyzed in the present study was compared to skin samples obtained from donkeys from the USA and controls (*B. besnoiti*, *B. tarandi*)

## Discussion

To the best of our knowledge, this is the first report of a series of cases of besnoitiosis in donkeys in the UK and the first time that the presence of *B. bennetti* infection in donkeys in Europe has been confirmed by microsatellite typing. Clinical besnoitiosis, typically characterized by weight loss, malaise and generalized dry skin, has not been observed in the cases reported herein. On the contrary, clinical presentation in the UK, of the *ante-mortem* cases, has chiefly been that of small skin masses with one case of ocular scleral besnoitiosis. Where lesions were confined to small nodular intradermal masses, there was limited health impact on the donkeys concerned. The first *B. bennetti* cases were identified incidentally as the skin masses were presumed to be sarcoid neoplasms. This is relevant with respect to UK practitioners as *Besnoitia* cysts should now be included as a differential diagnosis for sarcoids, at least in donkeys. This has implications for the prognosis for the individual animal and may be relevant for insurance purposes. At present it cannot be certain whether *Besnoitia* infection is confined to the donkey population or whether it has not been identified yet in other equines in the UK. Further studies to establish whether the cysts are also present in the horse and pony populations are warranted.

In Europe, clinical cases of besnoitiosis have been reported in France [1] and in Spain based on clinical signs and histopathological detection of cysts [8]. A later serological survey in Spain combining ELISA and western blot detected antibodies against *Besnoitia* spp. in donkeys, mules and horses [16]. Two cases of besnoitiosis in donkeys were reported in Belgium where *B. bennetti* infection was suspected based on clinical, histological, serological and molecular tools [5]. Recently, serological evidence for *Besnoitia* sp. infection, based on IFAT and western blot analysis, was detected in 0.3% of 385 horses from Portugal [11]. However, the identity of the *Besnoitia* sp. was not confirmed by microsatellite typing in Europe yet. The typing based on ribosomal DNA (rDNA) of the cases in Belgium revealed a difference of only 0.53% (i.e. differences in 5 of 938 nucleotide positions) relative to sequences of *B. besnoiti* and *B. caprae* [5] and, the sequence was not compared to *B. tarandi*. The comprehensive analysis performed in our study, using two serological assays on four serum and one tissue fluid samples, and two molecular assays on one skin sample, including microsatellite typing, clearly showed that the parasite species detected in donkeys in the UK is *B. bennetti*. Of the 6 microsatellite markers examined, 5 were identical to those reported in North American *B. bennetti*, while one (Bt-7) showed a single more repeat than *B. bennetti* isolates from the USA [10]. Bt-21 with only 6 repeats differed clearly from findings in *B. tarandii* (23–24 repeats) or *B. besnoiti* (12–15 repeats), i.e. Bt-21 represents a marker that clearly separates *B. bennetti* form other *Besnoitia* spp. from ungulates [10]. As more DNA markers are obtained from diverse *B. bennetti* samples, molecular databases can be enriched to facilitate the elucidation of the parasite population genetic structure.

In this study, several of the donkeys had histopathology performed on tissue surgically excised from sites that had previously had masses removed from the same area (i.e. recurrence of masses in same body area). *Besnoitia* cysts were only detected in the second round of histopathology examination, which may mean that *Besnoitia* cysts were not present in the lumps examined at the time of the first surgery, or that *Besnoitia* cysts may have been present but were missed. Therefore, we suggest that one needs to be careful when performing histopathology to not miss *Besnoitia* cysts. Indeed, alongside the sarcoids there was often only one cyst that could be missed in routine histopathological examination. Therefore, correct identification of *Besnoitia* cysts in excisional skin biopsies taken from UK donkeys does have a clinical advantage for veterinarians providing a more accurate prognosis where a skin mass has been identified. It can no longer be assumed that skin masses are either neoplastic in origin or inflammatory, such as eosinophilic granulomas, for example. In this case series, there does appear to be a correlation between the presence of sarcoid neoplasms and the presence of *Besnoitia* cysts, but this has not been subjected to statistical analysis. It is not possible to know whether the presence of *Besnoitia* cysts exacerbates sarcoid development or whether sarcoid tissue predisposes for the establishment of *Besnoitia* cysts in the adjacent dermis, or it is incidental, due to the frequency of sarcoids in donkeys. Noteworthy in the histopathology of 2 cases diagnosed with chronic laminitis was the presence of *Besnoitia* cysts, associated with moderate inflammation, within the stratum lamellatum and laminar corium of the hoof, suggesting that *Besnoitia* cysts may have played a role in laminitis development. Earlier reports showed a correlation between besnoitiosis and laminitis in cattle [17, 18]. Therefore, although our finding may be incidental, considering the associated inflammation, it is believed that the infection could, at least in part, contribute to this chronic inflammation of the hoof laminae. Further studies are required to determine whether there is any association between comorbidity with *Besnoitia* and sarcoids, *Besnoitia* and ocular lesions, *Besnoitia* and laryngeal lesions and the relevance between presence of *Besnoitia* cysts within the laminar corium and chronic laminitis in donkeys.

Much about the epidemiology of *B. bennetti* remains unknown. *Besnoitia bennetti* has been thought of historically as an exotic parasite and donkey besnoitiosis has been considered as an emerging disease [11, 19]. This raises the question about the source of infection and possible route of transmission in donkeys in the UK. Since its discovery, the route of *B. bennetti* transmission has not been clearly defined. Attempts to identify the definitive host for *B. bennetti* have been unsuccessful, precluding researchers from elucidating the parasite’s life-cycle [7]. All the donkeys described in this case series were kept in large groups on sites within a limited geographical range in East Devon and Dorset in England. It is possible that some factors specific to this cohort (e.g. the potential for transmission by blood-sucking insect vectors) may have facilitated *B. bennetti* transmission in these donkeys, similar to *Besnoitia* infection in cattle [20, 21]. However, the role of insect vectors in the transmission of donkey besnoitiosis has yet to be identified. Given the large number of donkeys kept on these sites, and the low case incidence, it can be assumed that the transmission rate remains low. However, we cannot be certain that we have identified and diagnosed all cases so far. Further studies are required to identify potential perpetuating factors, such as underlying disease, coinfection, the presence of potential wildlife reservoirs, or climatic conditions.

In our study, the donkeys presenting with intradermal masses were treated using surgical excision. Excision appears to have been locally curative as to date, no recurrence of skin masses has been recorded at the original presenting site. However, as mentioned above, it remains unknown whether this excisional treatment has been curative on a whole animal basis as there may well be other cysts elsewhere that have not been identified. Likewise, there has been no recurrence of clinical signs of conjunctivitis in the case with ocular presentation; however, the presence of cysts, in areas of the conjunctiva that cannot be assessed, again cannot be excluded. The second case with ocular *Besnoitia* cysts, found at PM examination, had a lengthy history of corneal ulceration and conjunctivitis, and arguably the presence of *Besnoitia* cysts had a detrimental *ante-mortem* effect on this donkey. The third case with ocular cysts, found at PM, had an *ante-mortem* history of epiphora. This was not present in the eye affected by the *Besnoitia* cysts and was not associated with any inflammation. Therefore, it is unlikely that *Besnoitia* was the cause of ocular symptoms in this donkey. In cases where laryngeal cysts have been identified, peripheral inflammation has been found around them. Although clinical signs of laryngeal discomfort in these donkeys were not reported, it cannot be ruled out entirely. Ongoing monitoring and investigation of clinical cases is justified to further elucidate any negative impact the presence of *Besnoitia* cysts may have on the donkeys. Given the lack of effective chemotherapeutic treatment for besnoitiosis, further research is required to determine the optimal treatment regimen, if any, for *B. bennetti* infection affecting donkeys. In the cases presented here, it is apparent that symptomatic treatment of associated inflammation, is all that has been required.

## Conclusions

Our results provided new insights into the presence of *B. bennetti* in donkeys in the UK and raised awareness of besnoitiosis in donkeys in the UK, particularly as a differential diagnosis for skin masses including sarcoids. Given the relative proximity to other European countries, where clinical besnoitiosis is apparent, UK veterinarians should be aware of the potential for this disease and the pattern of clinical signs during clinical examination. More efforts to understand *B. bennetti* phylogenetic and genomic properties are needed. This will improve the understanding of the epidemiology and the spread of besnoitiosis in donkeys and other equids, which may ultimately impact clinical management of equine besnoitiosis.

## Data Availability

All data generated or analyzed during this study are included in this published article.

## Abbreviations

ELISA: Enzyme-linked immunosorbent assay
FITC: Fluorescein isothiocyanate
H&E: Haematoxylin and eosin
H_2_O_2_: Hydrogen peroxide
IFAT: Indirect fluorescent antibody test
PBS-T: Phosphate buffered saline-Tween 20
PBS-TG: Phosphate-buffered saline-Tween 20, fish gelatine liquid
NSAIDs: Nonsteroidal anti-inflammatory drugs
PVDF: Polyvinylidene fluoride
RT-PCR: Real-time polymerase chain reaction
SDS: Sodium dodecyl sulfate
TBS: Tris-buffered saline
